# Antioxidant and Antimicrobial Effect of Plant Essential Oils and *Sambucus nigra* Extract in Salmon Burgers

**DOI:** 10.3390/foods10040776

**Published:** 2021-04-04

**Authors:** Kristina Jonušaite, Petras Rimantas Venskutonis, Gines Benito Martínez-Hernández, Amaury Taboada-Rodríguez, Gema Nieto, Antonio López-Gómez, Fulgencio Marín-Iniesta

**Affiliations:** 1 Department of Food, Science & Technology, Kaunas University of Technology, Radvilėnų pl. 19, LT-50254 Kaunas, Lithuania; kristina.jonusaite@ktu.lt; 2 Food Safety and Refrigeration Engineering Group, Department of Agricultural Engineering, Universidad Politécnica de Cartagena, Paseo Alfonso XIII 48, 30203 Cartagena, Murcia, Spain; GinesBenito.Martinez@upct.es (G.B.M.-H.); antonio.lopez@upct.es (A.L.-G.); 3 Department of Food Technology, Nutrition and Food Science, Veterinary Faculty, University of Murcia, 30100 Murcia, Spain; ataboada@um.es (A.T.-R.); gnieto@um.es (G.N.)

**Keywords:** *Sambucus nigra*, lipid peroxidation, antimicrobial, plant essential oil, salmon burger

## Abstract

The antioxidant capacity of oregano (OEO) and clove (CLEO) essential oils and black elderberry (*Sambucus nigra*) flower extract (SNE) were compared with butylhydroxytoluene (BHT) regarding its protection against lipid peroxidation and microbial counts in salmon burgers stored at 4 °C for 14 days and after cooking. The content of total phenols was 5.74% in OEO, 2.64% in CLEO and 2.67 % in the SNE. The total phenolic content and the antioxidant capacity were significantly higher (*p* < 0.05) for SNE and OEO. Both essential oils showed a similar IC_50_ and inhibition percentage of lipid peroxidation to BHT. The combination of OEO and SNE reduced 29% of thiobarbituric acid reactive substances (TBARS), while BHT reduced 31% of TBARS generated during refrigeration storage in salmon burgers in relation to the control sample without antioxidants. Additionally, the microbial counts after 14 days of refrigeration were the lowest in burgers when the combination of OEO and SNE was used. This study concludes that OEO and SNE can be used as inhibitors of lipid oxidation in salmon products and as natural candidates to replace commonly used synthetic antioxidants and antimicrobials in these food products.

## 1. Introduction

Foods, particularly red meat, fish and their derivatives, contain high amounts of pro-oxidant substances that cause the oxidation of polyunsaturated fatty acids (PUFAs). This oxidation process generates numerous degradation molecules, including malonyldialdehyde, which consequently alter the organoleptic and nutritional characteristics of food products [1,2].

Microbial contamination is the main cause of food spoilage, while oxidation processes are the second cause of alteration. Then, the addition of antioxidant compounds may affect these oxidations. The food industry has preferably used synthetic antioxidants such as butylated hydroxyanisole (BHA), butylated hydroxytoluene (BHT) and tertiary butylhydroquinone (TBHQ) because of the potential to inhibit the oxidation processes and the ability to do not modify food flavor.

BHA is effective in inhibiting lipid oxidation in ground beef at 1% [3], but the addition of synthetic antioxidants is restricted by most of the current regulations both regarding their doses and products. For instance, the use of gallates, TBHQ and BHA, in the category of heat-treated meat products, is only allowed for dehydrated meat [4]. The Food and Drug Administration (FDA) has established a limit of 0.02% of gallates because of suspicions of some toxic and possible carcinogenic effects associated with prolonged ingestion [5,6,7].

The research in relation to antioxidants from natural sources that ensure the safety of these additives is a current purpose [8]. The antioxidant activity of these substances comes mainly from their phenolic content: flavonoids, isoflavones, flavones, anthocyanins, catechins and other phenolic compounds [9]. Some extracts and essential oils from plants, such as citrus peel, rosemary (*Rosmarinus officinalis* L.), oregano (*Origanum vulgare* L.), pomegranate (*Punica granatum*), yerba mate (*Ilex paraguariensis*) and others, have been studied to test their antioxidant capacity in meat and fish products [2,10,11,12].

The use of an ethanolic extract of yerba mate in chicken meatballs has been reported as a good antioxidant, which decreased the lipid peroxidation based on the thiobarbituric acid reactive substances (TBARS) analysis and vitamin E depletion [13]. The addition of green tea (*Camelia sinensis*) at 0.25% substantially reduced the oxidation process in frozen mackerel. Additionally, the use of 2% rosemary extract was effective in controlling the lipid peroxidation and improving the sensory qualities in raw or cooked sardine fillets vacuum packed. Cold-water fish such as mackerel, salmon, tuna and sardines are more susceptible to rancidity because of their high PUFA contents. The use of effective natural antioxidants prevents the oxidation of fats and, therefore, improves the product preservation without modifying the organoleptic properties of the product [14,15].

Black elderberry (*Sambucus nigra*) has been studied to be used in ecological medicine for its pharmacological properties and its phenolic content [16,17,18]. The antioxidant properties of alcoholic extracts of the leaves, fruits and flowers of *S. nigra* have been associated with their high total flavonoid, linoleic acid and β-carotene contents [19,20]. The aim of this paper is to characterize the antioxidant properties of clove (CLEO) and oregano (OEO) essential oils, *S. nigra* flower extract (SNE) and their combination to improve the quality of salmon burgers by inhibiting the lipid oxidation and keeping acceptable sensory properties with an extended shelf life. The antioxidant and antimicrobial properties are assessed by comparison with the synthetic additive BHT, which is commonly used to avoid lipid oxidation in these food products.

## 2. Materials and Methods

### 2.1. Materials and Reagents

Folin–Ciocalteu reagent, BHT, 2-thiobarbituric acid (TBA), 1,1,3,3-tetraethoxypropane, 2,2-diphenyl-1-picrylhydrazyl stable radical (DPPH•) and 3,4,5-trihydroxybenzoic acid (gallic acid) were all of analytical grade and purchased from Sigma-Aldrich (Steinheim, Germany).

### 2.2. Plant Extracts

Black elderberry (*S. nigra*) flowers were purchased from local stores of organic products and medicinal herbs in Mažeikiai, Lithuania. In a previous paper, an example of the fractionation of *S. nigra* flowers into several products by using hydrodistillation and high-pressure extraction techniques was presented [21,22]. The extract that showed better antioxidant properties was the obtained using *S. nigra* flowers treated with SFE-CO_2_ and later by pressurized liquid extraction (PLE) with ethanol. Due to that, it was selected for the preservation of salmon burgers.

The optimal parameters for SFE-CO_2_ were defined (T = 50 °C, *p* = 45 MPa and v = 2 L/min), and the extraction was performed from 50–60 g of elderflower powder in these conditions. The residue was collected in light glass jars and used for further extraction with ethanol by PLE. This extraction process (PLE) was performed in a Dionex ASE350 apparatus (Dionex Corp., Sunnyvale, CA, USA). The obtained residue of SFE-CO_2_ (25 g) was extracted with 96% ethanol applying 2 extraction cycles at 60 °C, 5 min each. The solvent was removed at 150 ± 20 mbar pressure and 40 °C. The dark green gummy extracts were collected into dark glass vials and stored in a refrigerator until used.

The essential oils OEO and CLEO were obtained from the company Destilería Muñoz Gálvez Inc. (Murcia, Spain) by single-steam distillation. The main composition and the majority components of both were determined by Gas Chromatography with Flame-Ionization Detection. For CLEO, 81 different components were detected, and three of them represent 96.6% (1.9% α-caryophyllene, 17.8% β-caryophyllene and 76.9% eugenol). For OEO, 117 different components were detected, and nine of them represent the 93.5% (1.3% 1.8-cineol, 1.3% borneol, 3.1% g-terpinene, 4.3% linalool, 5.0% *p*-cymene, 2.7% β-caryophyllene, 1.4% a-terpineol, 2.2% Thymol and 72.2% Carvacrol).

All selected essential oils (OEO and CLEO), *S. nigra* extract (SNE) and BHT were prepared in ethanol–water (6:4 volume (*v*:*v*) (E-W), so that the total concentration of antioxidant compounds (natural and/or synthetic) for each treatment was 0.01% in the burger dough.

Fresh salmon fillets were purchased from a local market in Murcia (Spain). The skin from fillets was removed, and the fillets were kept at 4 °C until the burger preparation (less than 24 h).

### 2.3. Preparation of Fish Burgers

The composition of salmon burgers was salmon meat (92.05%), salt (0.92%), dried garlic (0.09%), dried potato powder (6.93%) and an antioxidant mix (0.01%) (Table 1).

The fish meat was ground and mixed with the rest of the ingredients in a domestic meat mincer. This burger mass was divided into seven groups (~700 g per group; 10 burgers for each group): five treatments with the different antioxidants to be evaluated and two controls (see Table 1).

The prepared burgers (70 g) were formed with a metal mold 7.45 cm in diameter and 1.5 cm deep. A plastic film was used to avoid cross-contamination between groups and to avoid deformation of the hamburgers when they were removed from the mold. The burgers were vacuum-packed in high-density polyethylene bags and stored at 4 °C for 14 days. Samples were taken at the initial time (day 0) and after 14 days of storage for analysis. Cooking of burgers was carried out by grilling in a pan to ensure 70 °C in the center of burgers in accordance with preliminary experiments.

### 2.4. Total Phenols and Antioxidant Activity of S. nigra Flower Extract (SNE) and Essential Oils of Clove and Oregano (OEO and CLEO)

The total phenolic contents of SNE, OEO and CLEO were determined using the Folin–Ciocalteu method [23] with slight modifications as follows. Aliquots (1 mL) of each SNE, OEO or CLEO were diluted 1:10 in methanol:water (6:4 *v*:*v*) and centrifuged (10 min, 756× *g*). The diluted sample (125 μL) was mixed with 125 μL of Folin–Ciocalteu reagent, and 1.25 mL of saturated sodium carbonate solution (7%) was added. Finally, 1.3 mL of distilled water was added to the mixture and shaken gently in a vortex. After 90 min of incubation in darkness (room temperature), the absorbance was measured at 760 nm in triplicate using a UV–VIS spectrophotometer (Nicolet evolution 300, Thermo Fisher Scientific, Madrid, Spain). A calibration curve was prepared by using a standard solution of gallic acid (0–600 μg/mL), and the results of phenols were expressed as mg gallic acid equivalent per 100 mL.

The scavenging capacity of samples against DPPH radical [24] was assessed according to the method of Blois [25], with some modifications. An aliquot (2.5 mL) of the diluted sample (1:10 in methanol) was mixed with 0.5 mL of DPPH• solution (1 mM prepared in methanol). The reaction mixture was vortexed thoroughly and left in the dark at room temperature (25 ± 1 °C) for 30 min. The decrease in the absorbance (due to the electron/proton donating activity) was measured at 517 nm using the UV–VIS spectrophotometer. The results are expressed as the concentration (g/100 mL) of essential oils or SNE, which reduces the concentration of radicals (IC_50_) by 50%.

The ability to scavenge DPPH• (I) was calculated by the following equation:

(I) = [(A_0_ − A_1_)/A_0_] (1)

where A_0_ is the absorbance of the control at 30 min, and A_1_ is the absorbance of the sample after 30 min. Samples were analyzed in triplicate.

### 2.5. Fish Burger Analyses

#### 2.5.1. pH and Water Activity (*a_w_*)

The measurement of pH was carried out on 10 g of uncooked samples, homogenized in distilled water (1:10 sample (*weight*):water (*v*)) using a pH meter (Crison GLP 21, Crison Instruments S.A., Barcelona, Spain).

The water activity (*a_w_*) values were determined in uncooked salmon burgers using a Novasina LabSwift-*a_w_* (Lachen, Switzerland).

#### 2.5.2. Color Determination

The color surface of uncooked burgers was determined by using a colorimeter (Konica Minolta CM-2600d/2500d, Osaka, Japan) based on the CIELab parameters (L*, a* and b*) [26]. Due to the color surface variation, 30 measures were performed on each burger. The values were processed by the Spectra Magic software (version 2.11, Minolta Cyberchrom Inc., Osaka, Japan).

#### 2.5.3. Microbial Analysis of Burgers

Samples of 10 g of burger were aseptically weighed and homogenized with 90 mL of 0.1% sterile peptone water in a masticator blender (IUL Instruments, Barcelona, Spain) for 1 min at room temperature. Serial decimal dilutions were prepared for each sample in 0.1% peptone solutions (Merck, Darmstadt, Germany), and 1 mL of the samples in appropriate dilutions, in duplicate, were poured and spread for total count and selective agar plates, respectively. Total viable counts of psychrotrophic microorganisms [27], coliforms [28], molds, yeasts [29] and lactic acid bacteria (LAB) [30] were determined. The results are expressed as the log difference between counts after 14 days of storage (4 °C) (Nf) and initial counts in burger mass (Ni).

#### 2.5.4. Inhibition of Lipid Peroxidation. Thiobarbituric Acid Reactive Substances (TBARS)

For the seven treatments of burger dough with different antioxidant composition were prepared three groups of burger samples for analysis, uncooked burgers, before refrigeration storage (0 days) and after 14 days of refrigeration storage and cooked burgers after 14 days of storage. The samples were processed by taking 5 g of them mixed with 15 mL of distilled water in a Brinkman Polytron homogenizer (PT type 10/35, Westbury, NY, USA) during 30 s at 16,000 rpm. The homogenized samples were kept on ice and then were centrifuged at 1344× *g* for 10 min. The supernatant was separated and stored at −20 °C until used for analysis. The TBAR determination was conducted as described by Silbande et al. (2016) [31]. Briefly, 5 mL of the supernatant was added to 5 mL of 0.02 mol/L TBA, heated in a boiling water bath for 35 min and then immediately cooled to room temperature in ice. The absorbance was measured at 532 nm using the UV–Vis spectrophotometer. The TBARs value was calculated from a standard curve of malondialdehyde (MDA), which was freshly prepared by the acidification of 1,1,3,3-tetraethoxypropane (TEP). TBARs values were expressed as mg MDA/kg of burger. TBARS was calculated in cooked and uncooked samples.

#### 2.5.5. Sensory Analysis

The sensory assessment was performed by 25 panelists (21 women and 4 men) who were recruited from students and employers of the Faculty of Veterinary of the University of Murcia (Spain). The students received 7 samples (Table 1) of the salmon burgers immediately after cooking and stored in refrigeration after 14 days of storage.

The burger samples (70 g) were coded with a randomly selected three-digit number; afterwards, all samples were cooked in the same conditions and immediately presented in random order to the panelists. The panelists were installed in individual sensory booths under artificial white light, and the samples were presented in white dishes coded with three-digit random numbers. Each judge was provided with a glass of water and unsalted crackers to cleanse the palate between samples. The sensory test involved an evaluation for the acceptability of the samples in which panelists judged the color, odor, flavor and overall acceptability of the samples in accordance with a nine-point hedonic scale (1 = dislike extremely to 9 = like extremely) [32].

### 2.6. Statistical Analysis

The statistical significance of differences among treatment means was evaluated by analysis of variance (one-way ANOVA), and the means were compared using Tukey’s test with significance at *p <* 0.05. Data were evaluated statistically using the SPSS version 13.0 for Windows (SPSS, Chicago, IL, USA).

## 3. Results

### 3.1. Total Phenols and Antioxidant Activity in S. nigra Flower Extract (SNE) and Essential Oils of Clove and Oregano (OEO and CLEO)

**Figure 1:**
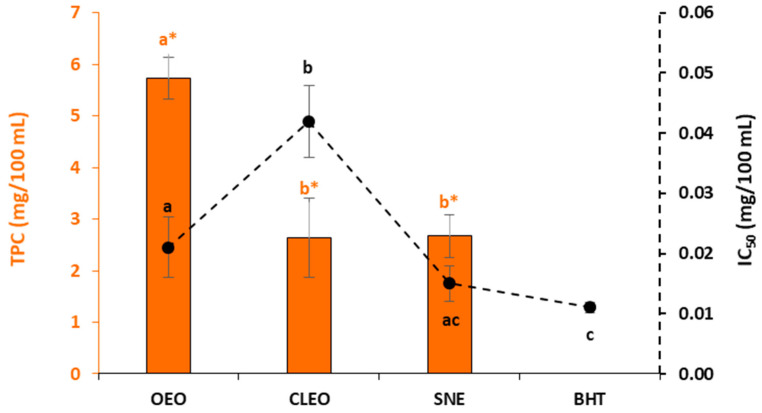
Total phenolic content (TPC) (■) and antioxidant capacity expressed as IC_50_ values of the 2,2-diphenyl-1-picrylhydrazyl (DPPH)▪ scavenging activity (●) in oregano essential oil (OEO), clove essential oil (CLEO), *S. nigra* flower extract (SNE) and butylhydroxytoluene (BHT) (mean ± SD). Different letters mean significant differences (orange letters with asterisks for the TPC and black letters without asterisks for the IC_50_ values).

The OEO presented the significant highest Folin–Ciocalteu assay value, which is used in this study as indicative of the total phenolic content (Figure 1). It was significantly different from CLEO and SNE, which did not show differences between them in terms of the total phenolic content (*p <* 0.05).

The values of the scavenging effect of the radical DPPH are shown in Figure 1, expressed as IC_50_ values. A lower IC_50_ value indicates a higher antioxidant activity of samples. The best free-radical scavenging activity was obtained with BHT. OEO demonstrated a lower antioxidant activity than BHT and SNE but did not show significant differences with SNE, while CLEO showed the lowest antioxidant capacity (*p* < 0.05).

### 3.2. Fish Burger Analysis

#### 3.2.1. pH, Water Activity (*a_w_*) and Color

The pH values of burgers after 14 days of storage at 4 °C are shown in Table 2. The initial pH of burgers was 5.82 (data not shown). There were no significant pH differences (*p* > 0.05) after 14 days, except for the positive control (without antioxidants) that increased up to 6.10.

On the other hand, the *a_w_* did not present significant changes between samples at the beginning of the experiment nor during storage. Thus, the *a_w_* values ranged between 0.948 and 0.951, without significant differences among samples (*p* > 0.05).

The initial values of L* (lightless), a* (redness) and b* (yellowness) were 34.29–40.39, 7.69–10.98 and 13.09–15.94, respectively (Table 2). These values were similar to those obtained at the beginning of the experiment. The positive control sample showed higher values of L* and b*, and the lowest of a*, although significant differences were not found compared with the other treatments (*p* > 0.05).

#### 3.2.2. Microbial Counts

**Figure 2:**
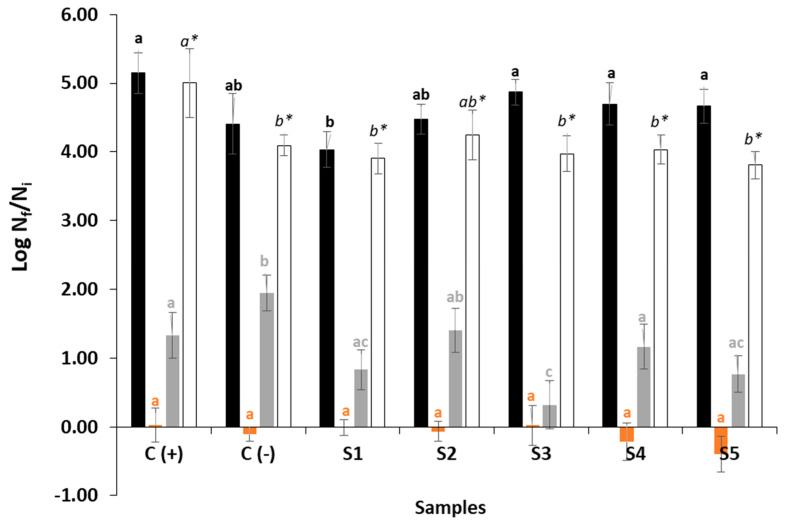
Microbial count increase during 14 days of refrigeration storage of salmon burgers. Total viable count of psychrotrophic microorganisms (■, black letters), molds and yeasts (■, orange letters), lactic acid bacteria (LAB) (■, gray letters), coliforms (□, *italic* and asterisk *). See Table 1 for details of the antioxidant composition in burger samples. Different letters mean significant differences between groups of microorganisms (mean ± SD).

In accordance with the results obtained in this study, the antimicrobial effects of the studied plant essential oils (OEO and CLEO) and the *S. nigra* extract (SNE) may be due to the antimicrobial activity of the phenolic compounds present. In this study, the combination of SNE and OEO (sample S5) showed both, the highest concentration of phenolic compounds and the best antimicrobial effect in salmon burgers after 14 days under refrigeration (Figure 2). In fact, S5 samples showed good control of all the microbiological values, especially of molds and yeasts, where the greatest decrease was obtained concerning the values at 0 days, although the statistical analysis did not show significant differences between the samples. In addition, the S1 samples (SNE + CLEO) showed the lowest total viable counts of psychrotrophic microorganisms and, at the same time, a good control of coliforms, BAL and molds and yeasts.

#### 3.2.3. Inhibition of Lipid Peroxidation. Thiobarbituric Acid Reactive Substances (TBARS)

**Figure 3:**
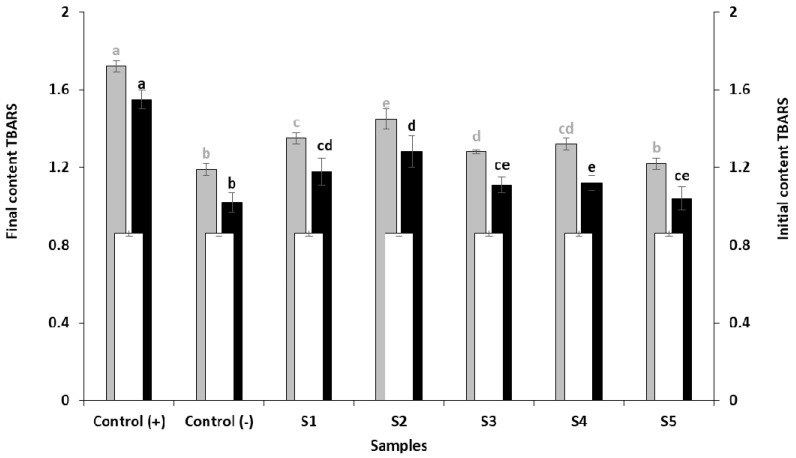
Thiobarbituric acid reactive substances (TBARS) content (mg malondialdehyde (MDA)/kg) in salmon burgers at the beginning of the experiment (□) after refrigeration storage at 4 °C (■, gray letters) and after refrigeration storage at 4 °C and cooking (■, black letters). See Table 1 for details of the antioxidant composition in burger samples. Different letters mean significant differences between burger groups before and after cooking (mean ± SD).

Figure 3 shows the evolution of the TBAR values of salmon burgers after 14 days of refrigeration and after cooking. The TBARS value was of 0.86 mg MDA/kg at the beginning of the experiment.

The TBAR values in the negative control and S5 (SNE + OEO) sample were 1.19 and 1.22 MDA/kg, respectively, which were significantly lower than the other treatments (*p <* 0.05) after storage at 4 °C. The lipid oxidation in the positive control sample showed the highest value of TBARS (1.72 MDA/Kg) with significant differences with the other samples. The S1 samples (SNE + CLEO) and S4 (OEO) presented similar values (1.35 and 1.32 MDA/kg); they were significantly different from S2 samples (Cl, 1.45 MDA/kg). S3 (SNE, 1.28 MDA/kg) was different from S2 but not from S4 (OEO, 1.32 MDA/kg).

#### 3.2.4. Sensory Analysis

The results of the sensory evaluation of the salmon burgers, control and samples treated with natural antioxidants did not show significant differences (*p* < 0.05) for the parameters of color, odor, flavor and general acceptability (Table 3). All samples were well-accepted, although no significant differences were observed between them. It can be also highlighted that the sample without antioxidants (control +) was the worst valued by the panelists, as shown in Table 3.

## 4. Discussion

In this study, the phenolic content and the antioxidant activity of CLEO and OEO essential oils and *S. nigra* flower extract were studied before the application of these natural compositions to salmon burgers to inhibit lipid peroxidation and microbial growth. The antioxidant activity of plant essential oils is related to the capacity of polyphenols to act as metal chelators, free-radical scavengers, hydrogen donators and inhibitors of the enzymatic systems responsible for initiating the oxidation reaction. Furthermore, they can act as a substrate for free radicals like superoxide or hydroxyl or intervene in propagation reactions [33,34].

The contents of phenolic compounds of the essential oils and the flower extracts studied in this work revealed that these contain many bioactive compounds, which gives them a high antioxidant capacity. Regarding phenolic compounds, *S. nigra* has been reported as a plant material with high content of flavonoids (rutin and quercetin) and phenolic acids (gallic acid and gentisic acid) [20]. The main compounds of CLEO and OEO were eugenol (76.9%) and carvacrol (72.2%), respectively. This fact has been reported by other authors, which found that eugenol is a remarkably better antioxidant in comparison to carvacrol [35]. Stoilova et al. [36] found SNE as a better antioxidant than BHT because the elderflower extract showed a significantly greater antiradical activity concerning DPPH scavenging, although, in our results, there were no significant differences between them. Free-radical scavenging is one of the known mechanisms able to inhibit lipid oxidation. It is a rapid and widely used method to characterize the antioxidant activity of plant materials [37]. Several studies have evaluated the relationship between the antioxidant activity of plant products and their phenolic content. Substances that can perform this reaction can be considered as antioxidants and radical scavengers [38]. This study indicates that higher total phenol contents lead to better DPPH scavenging activity of the antioxidants studied, and this agrees with previous reports [39].

Different groups of burgers were prepared using different combinations of the natural antioxidants mentioned above, and the results after refrigerated storage (4 °C, 14 days) were compared with BHT, a common additive used to avoid lipid peroxidation in food products.

Several authors have reported different results on the decrease or increase of pH in various species of fish stored at refrigeration temperatures [40,41,42]. Alkaline substances produced from bacteria, such as ammonia, were likely to increase this pH value, and the accumulation of lactic acid produced by glycolysis could decrease this parameter [43]. The observed results in this paper indicate that the plant bioactive products used as antioxidants stabilize the pH value in salmon burgers. Furthermore, the synthetic antioxidant (BHT) in the negative control samples had a similar behavior. The pH range of the salmon burger samples of this work is similar to that obtained in other studies conducted with salmon meat [40].

Significant *a_w_* differences were not detected between samples. The hereby observed *a_w_* range is lower than the values reported for salmon meat in the literature [44]. However, the composition of burger dough could influence this *a_w_* diminution mainly due to the salt used as an ingredient in the formulation.

The results of color characterization could indicate a trend toward a color variation. The color of salmon muscle can be attributed to the astaxanthin carotenoid, as well as to the haem pigments [40,45]. The decrease of luminosity in the salmon burger samples with antioxidants could be due to the oxidation of these compounds (negative control and S1-S5). The antioxidants perform a protective effect, because they are preferentially oxidized to lipids. The color results in this study, in positive control samples in comparison to the samples treated with antioxidants, has been also previously observed in tuna fillets: the loss of red color occurred in parallel with TBARS development during cold storage of tuna fillets [46].

In accordance with microbial counts of burger samples stored under refrigeration (4 °C) during 14 days, the lower counts were detected in samples containing SNE (S1, S3 and S5). Nevertheless, significant differences were not generally found between the burger groups. The SNE was the natural extract that showed the highest antioxidant activity (Figure 1). The content of phenolic compounds has been correlated with the antimicrobial activity in previous studies, which reported that the hydroxyl groups of polyphenols can interact with the cell membrane of bacteria and cause their destruction. Additionally, the antimicrobial activity is influenced by the different molecular structure of phenols [47]. In this study, a direct relationship between the total phenolic content and antimicrobial activity was found in accordance with the total phenolic content of OEO and SNE. Similar findings were observed for the antioxidant activity and antimicrobial capacity of the antioxidant compositions studied (Figure 1 and Figure 2).

Our observations indicated that *S. nigra* extract and OEO and CLEO are potential candidates to limit the lipid oxidation in salmon burgers. Similar findings were found with oregano extracts applied to restrain lipid oxidation in raw pork [48]. Grape seed extracts were also found to inhibit lipid oxidation in rainbow trout meat [49], while sage extracts reduced lipid oxidation in porcine liver paste [50]. The observed antioxidant effects in salmon burgers are mainly influenced by the use of SNE and/or OEO. The use of CLEO (S2) or OEO (S4) showed significant differences, the combined effect of SNE and OEO was significantly better than the use of SNE and CLEO. This effect is not consistent with the antioxidant activity of the essential oils studied previously in the lab analysis and the antioxidant capacity reported for the majority of compounds of CLEO (eugenol) and OEO (carvacrol) [35]. The observed results led to thinking in some interference occur when CLEO are applied in salmon burgers, and some enhancing effect takes place for OEO. It must be highlighted that all the treatments significantly improved the results concerning the lipid oxidation inhibition in comparison to control without antioxidants (positive)

The high content of phenolic compounds in OEO and SNE (5.74% and 2.67% gallic acid equivalents respectively), as well as the possible synergism of its antioxidant qualities in general, could have led to an increase in the protection of PUFAs in salmon meat. This effect was similar to that induced by BHT, one of the most widely used chemical antioxidants for this purpose in the food industry. The antioxidant effect of elderflower extract and BHT has been compared before, and the capacity of *S. nigra* as an antioxidant has been demonstrated [36]. From our results, it can be inferred that the mixture of OEO and *S. nigra* extract could be adequate to retard the oxidation of lipids in salmon meat.

TBARS values indicate the content of lipid oxidation byproducts, primarily aldehydes (or carbonyls) that contribute to a rancid taste in meat and fish products [51]. In our study, the TBARS values increased in all samples during storage, mainly in the positive control samples with an increase of 0.86 mg of TBARS at the end of the storage period. However, the smallest TBAR increases corresponded to salmon burgers treated with BHT (0.33 mg) and the OEO-SNE mixture treatment (0.36 mg). These results show that the protective effect using the mixture OEO-SNE was effective to preserve PUFAs of salmon burgers, as well as the use of BHT as a synthetic antioxidant. Our findings are in accordance to Pakawatchai et al. [45], who obtained a significant antioxidant effect with the use of pepper and garlic paste in minced salmon meat stored at 4 °C for 12 days. Furthermore, the combination of rosemary, ascorbic acid and alpha tocopherol has shown higher protection against rancidity in ostrich fillets than the use of individual antioxidants during storage at 4 °C for 21 days [52]. Our results agree with others [53], which used alcoholic extraction to obtain SNE. These authors report that the main group of phenolic compounds found in *S. nigra* flowers with high antioxidant capacity are hydroxycinnamic acids, flavonols and flavanols. In this review is also reported that *S. nigra* flowers, usually have higher antioxidant activity than berries and leaves, and that SNE has better antioxidant properties than synthetic antioxidants as BHT for inhibiting the lipid oxidation in foods. It is important to highlight that the pan grilling of burgers caused the loss of a significant amount of oxidation products formed during the storage period, with the loss of 0.17 mg in most of the treatments (Control (+), Control (−), S1, S2 and S3) and 0.20 and 0.18 mg for OEO and OEO + SNE, respectively. It suggests that these losses are mainly due to thermal degradation rather than the action of a particular antioxidant applied.

In the present study, the sensory evaluation was consistent with the TBAR values in the samples containing the antioxidants. The phenolic composition of the *S. nigra* extract and the used essential oils (OEO and CLEO) were effective to minimize the lipid oxidation in salmon burgers. Therefore, these plant bioactive products could be used in salmon burgers due to the observed high antioxidant properties. Color, odor and flavor alterations are considered very important quality attributes in meat products that are largely related to the degree of PUFA oxidation in this type of food. In accordance with the assessment received by burgers kept for 14 days in refrigeration and the best results in terms of lipid oxidation (TBARS), it can be stated that *S. nigra* extract and OEO, and their combination, can be used to retard the oxidative rancidity in salmon burgers, without altering their organoleptic properties.

## 5. Conclusions

In accordance with the results obtained in this study, the antimicrobial and antioxidant effects of essential oils of oregano and clove and the *S. nigra* flower extract may be due to their high phenolic contents. These compounds have shown a protective effect against microbial growth and the oxidation of lipids in salmon burgers. Our observations indicate that there is a direct relationship between the content of the polyphenols and the inhibition of the oxidation of PUFAs in burgers during storage at 4 °C for 14 days. A similar decrease in the TBAR content was detected after cooking in all the samples, which indicates that the thermal effect was responsible for this diminution. The use of oregano essential oil, together with the *S. nigra* extract, demonstrated an antioxidant capacity very similar to that achieved with a common synthetic antioxidant (i.e., BHT), with similar contents of antioxidants in the burgers (0.1%). Additionally, the antimicrobial effect of this combination of natural antioxidants in burgers was the highest after 14 days of refrigeration.

The sensory evaluation did not show significant differences between the different treatments, and all the samples were evaluated positively. No significant changes were detected for the color, odor or flavor of burgers, as well as odors and flavors attributable to the typical rancidity due to the oxidation of lipids. Although no significant differences were detected, the samples without antioxidants were those that received the lowest score for all the organoleptic attributes compared. Consequently, the results obtained in this study show that oregano essential oil and *S. nigra* extract can be used as inhibitors of lipid oxidation and microbial growth in salmon products and could replace the synthetic antioxidants commonly used in this kind of food.

## Figures and Tables

**Table 1:** Antioxidant mix composition in burger samples (0.01%). Butylhydroxytoluene (BHT). *Sambucus nigra* flower extract (SNE), clove essential oil (CLEO) and oregano essential oil (OEO), all of them prepared in ethanol:water (6:4, *v:v*) (E-W).

Samples	E-W (%)	BHT (%)	SNE (%)	CLEO (%)	OEO (%)
Control (+)	0.01	-	-	-	-
Control (−)	-	0.01	-	-	-
S1	-	-	0.005	0.005	-
S2	-	-	-	0.01	-
S3	-	-	0.01	-	-
S4	-	-	-	-	0.01
S5	-	-	0.005	-	0.005

**Table 2:** pH, water activity (*a_w_*) and color of raw salmon burgers (mean ± SD). See Table 1 for details of the antioxidant composition in burger samples. Different letters mean significant differences for each parameter between burger groups after 14 days of storage at 4 °C.

Samples	*a_w_*	pH	COLOR		
			L*	a*	b*
C (+)	0.948 ± 0.002 ^a^	6.10 ± 0.03 ^a^	40.39 ± 10.78 ^b^	7.69 ± 3.54 ^b^	15.94 ± 2.42 ^b^
C (−)	0.949 ± 0.003 ^a^	5.81 ± 0.03 ^b^	34.96 ± 7.32 ^b^	10.78 ± 3.55 ^b^	13.09 ± 4.47 ^b^
S1	0.950 ± 0.003 ^a^	5.84 ± 0.03 ^b^	35.55 ± 6.25 ^b^	10.98 ± 2.59 ^b^	12.75 ± 4.37 ^b^
S2	0.951 ± 0.002 ^a^	5.86 ± 0.02 ^b^	34.78 ± 6.75 ^b^	10.55 ± 4.57 ^b^	13.57 ± 3.47 ^b^
S3	0.949 ± 0.003 ^a^	5.82 ± 0.03 ^b^	34.45 ± 8.11 ^b^	10.57 ± 3.55 ^b^	13.72 ± 5.47 ^b^
S4	0.951 ± 0.002 ^a^	5.84 ± 0.03 ^b^	34.87 ± 9.21 ^b^	11.03 ± 5.52 ^b^	13.25 ± 6.53 ^b^
S5	0.950 ± 0.002 ^a^	5.83 ± 0.02 ^b^	34.29 ± 9.18 ^b^	10.58 ± 4.54 ^b^	13.73 ± 2.57 ^b^

**Table 3:** Sensory evaluation. See Table 1 for details of the antioxidant composition of the salmon burger (mean ± SD). Different letters mean significant differences between cooked burger groups for each sensorial attribute.

Samples	Color	Odor	Flavor	Overall Acceptability
C (+)	6.8 ± 0.79 ^a^	6,02 ± 1.03 ^a^	6.7 ± 0.82 ^a^	6.7 ± 0.82 ^a^
C (−)	7.5 ± 0.71 ^a^	7.5 ± 1.08 ^a^	7.6 ± 0.97 ^a^	7.7 ± 0.82 ^a^
S1	7.2 ± 0.92 ^a^	7.3 ± 0.67 ^a^	7.5 ± 0.71 ^a^	7.3 ± 0.82 ^a^
S2	7.1 ± 1.1 ^a^	6.8 ± 0.79 ^a^	7.2 ± 0.79 ^a^	7.0 ± 1.05 ^a^
S3	7.1 ± 0.99 ^a^	7.1 ± 0.57 ^a^	7.3 ± 0.67 ^a^	7.2 ± 0.79 ^a^
S4	7.2 ± 0.92 ^a^	6.9 ± 0.99 ^a^	7.3 ± 0.95 ^a^	7.1 ± 0.74 ^a^
S5	7.3 ± 1.16 ^a^	7.0 ± 1.05 ^a^	7.0 ± 0.67 ^a^	7.1 ± 0.88 ^a^

## Data Availability

The data presented in this study are available on request from the corresponding author.
